# Echinococcus Granulosus Infection in Two Free-Ranging Lumholtz’s Tree-Kangaroo (*Dendrolagus lumholtzi*) from the Atherton Tablelands, Queensland

**DOI:** 10.3390/tropicalmed3020047

**Published:** 2018-05-03

**Authors:** Amy L. Shima, Constantin C. Constantinoiu, Linda K. Johnson, Lee F. Skerratt

**Affiliations:** 1 One Health Research Group, College of Public Health, Medical and Veterinary Science (CPHMVS), James Cook University, Townsville, Queensland 4811, Australia; lee.skerratt@jcu.edu.au; 2 College of Public Health, Medical and Veterinary Science, James Cook University, Townsville, Queensland 4811, Australia; constantin.constantinoiu@jcu.edu.au; 3 University of Colorado-Denver, Aurora, CO 80045, USA; linda.k.johnson@ucdenver.edu

**Keywords:** echinococcus, hydatid disease, tree-kangaroo, zoonosis, public health

## Abstract

Infection with the larval stage of the cestode, *Echinococcus granulosus* sensu lato (s.l.)*,* causes hydatid disease (hydatidosis) in a range of hosts, including macropods and other marsupials, cattle, and humans. Wild macropods are an important sylvatic reservoir for the life cycle of *E. granulosus* (s.l.) in Australia, and so provide a conduit for transmission of hydatid disease to domestic animals and humans. Two Lumholtz’s tree-kangaroos (*Dendrolagus lumholtzi*) from the Atherton Tablelands of Far North Queensland were recently found to have hydatid cysts in both liver and lung tissues. Tree-kangaroos may travel across the ground between patches of forest but are primarily arboreal leaf-eating macropods. The finding of hydatid cysts in an arboreal folivore may indicate that the area has a high level of contamination with eggs of *E. granulosus* (s.l.). This finding may be of significance to human health as well as indicating the need for further investigation into the prevalence of hydatid disease in domestic stock, wildlife and humans living in this rapidly urbanizing region.

## 1. Introduction

Human echinococcosis is estimated to affect 2–3 million people worldwide with 84 to 89 new cases reported per year in Australia, yet it is a ‘neglected’ disease [1,2,3,4]. Human echinococcosis is caused by the cestodes *E. granulosus* (s.l.) (hydatid disease), *E. multiocularis* (alveolar hydatid disease), or *E. vogeli* and *E. oligarthus* (polycystic echinococcosis). *E. granulosus* (s.l.) is found throughout the world and canids are the definitive host. Hydatid cysts may develop in the lungs, liver, brain, or other internal organs of intermediate hosts such as humans, cattle, macropods, and other marsupials. Hydatidosis in Australia has been recognized since the 1860s. Transmission of *E. granulosus* (s.l.) in Australia has been facilitated by the presence of definitive hosts (dingoes and dogs) and naïve susceptible intermediate hosts such as macropods. Environmental conditions in eastern Australia, particularly in coastal areas and along the Great Dividing Range, with >25 mm/month rainfall for 6 months of the year, are favorable for transmission of the parasite [4].

The role of macropods as a sylvatic reservoir of the parasite has been well documented [5,6,7]. Jenkins [7] noted that hydatid cysts most commonly occur in the lungs of kangaroo and wallaby hosts with cysts in the liver occasionally seen in the eastern grey kangaroo, *Macropus giganteus*. Localization of cysts in pulmonary tissues of macropods such as rock wallabies likely compromises lung function and reduces fright and flight responses, making them susceptible to predation [8,9]. Banks [6] found that *E. granulosus* eggs in faeces deposited by dingoes tend to be concentrated on the verges of dense scrub where wallabies congregate, rather than in open woodland or savannah, and therefore increase the likelihood of transmission among hosts.

In this report, we describe hydatidosis in Lumholtz’s tree-kangaroos for the first time. Tree-kangaroos are an iconic representative of biodiversity in the Wet Tropics World Heritage Area of Australia and their predominantly arboreal existence makes infection with a parasite present on the ground unusual. Lumholtz’s tree-kangaroo is one of Australia’s largest arboreal marsupials [10], coming to ground primarily to move between patches of rainforest trees or while dispersing from the maternal home range (see Supplemental Figure S1). Due to fragmentation of habitat on the Atherton Tablelands, tree-kangaroos commonly travel between patches of forest and, as such may come into contact with dingo or wild dog faeces deposited at the interface between remnant forest and grasslands. As the eggs of *E. granulosus* can survive for many months in the environment and will be dispersed over large areas by wind, rain, and insects, animals will be exposed to infective eggs as they travel across the ground [3,11]. Lumholtz’s tree-kangaroo were among the 29 native prey species identified by Vernes and Dennis [12] consumed by dingoes on the Atherton Tablelands. Hence, participation in the life cycle of *E. granulosus* by Lumholtz’s tree-kangaroos and dingoes is possible. 

Injured and dead Lumholtz’s tree-kangaroo were collected from various locations on the Atherton Tablelands as part of a mortality study conducted between August 2012 and December 2017. These were collected through a concerted campaign utilizing the local wildlife care network, media, and social media. The public was requested to notify the senior author (ALS) by telephone of injured or dead tree-kangaroos. Upon being notified of a dead or injured tree-kangaroo, the senior author visited the site, confirmed host species identity, recorded site coordinates, and performed a post-mortem examination on the carcass to determine the cause of death. Tissue samples were collected for histopathology. Presence of *E. granulosus* was confirmed by histological identification of protoscolices in the ‘hydatid sand’ collected from cysts and brood capsules. Molecular diagnostics would have been useful to confirm the identity of the strain of *Echinococcus* but were not carried out due to a lack of resources.

## 2. Cases

### 2.1. Case #1: 4.6 Kg Female Lumholtz’s Tree-Kangaroo (Case 16-532)

A 4.6 kg female Lumholtz’s tree-kangaroo was killed by a dog on a cattle property on the Atherton Tablelands, QLD. The dog responsible for killing this tree-kangaroo was on a regular program of deworming, which included praziquantel. The tree-kangaroo carcass was intact and had not been fed upon. Based on lack of enlarged teats, active ovarian tissue and dentition, the tree-kangaroo was estimated to be a nulliparous female approximately 3 years old. 

**Figure 1:**
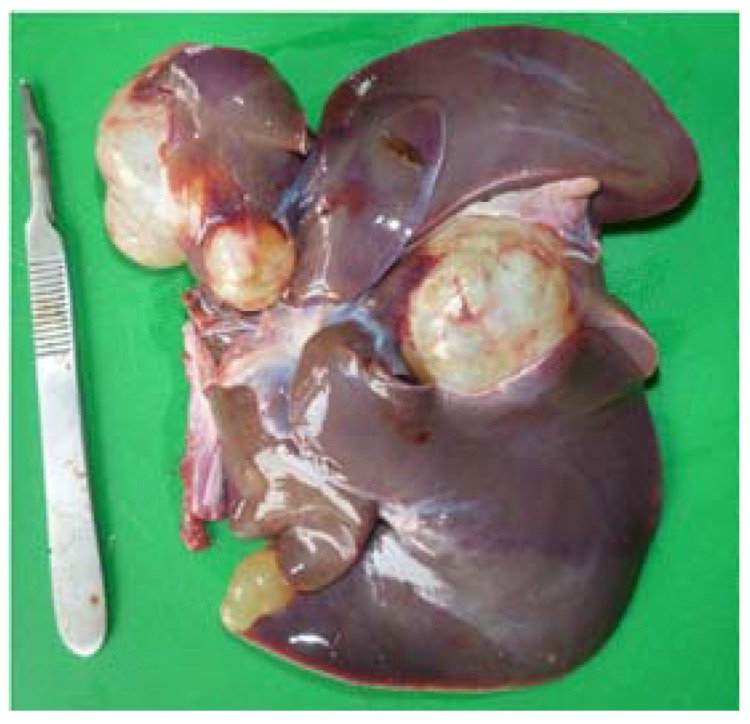
Liver with hydatid cysts. Photo: A. Shima.

On post-mortem examination, extensive bite wounds were found over the tail, head, and caudal abdomen. On opening the abdomen, large cystic structures were found occupying approximately a quarter of the liver mass (Figure 1).

**Figure 2:**
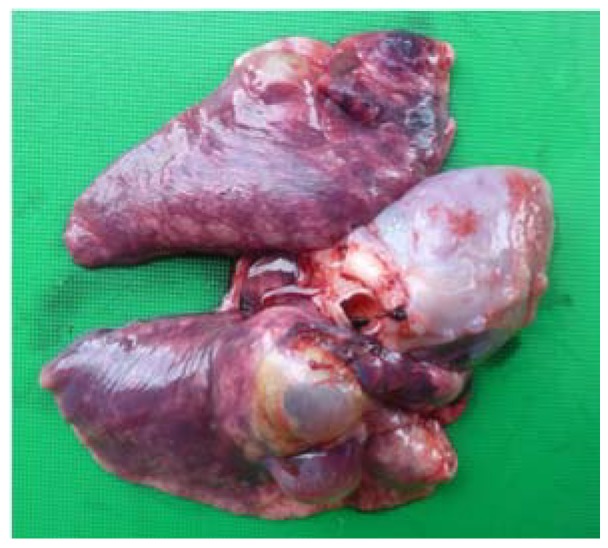
Lungs with hydatid cysts and heart. Photo: A. Shima.

Examination of the thoracic cavity revealed multiple cystic structures occupying approximately half of the total lung volume (Figure 2). 

**Figure 3:**
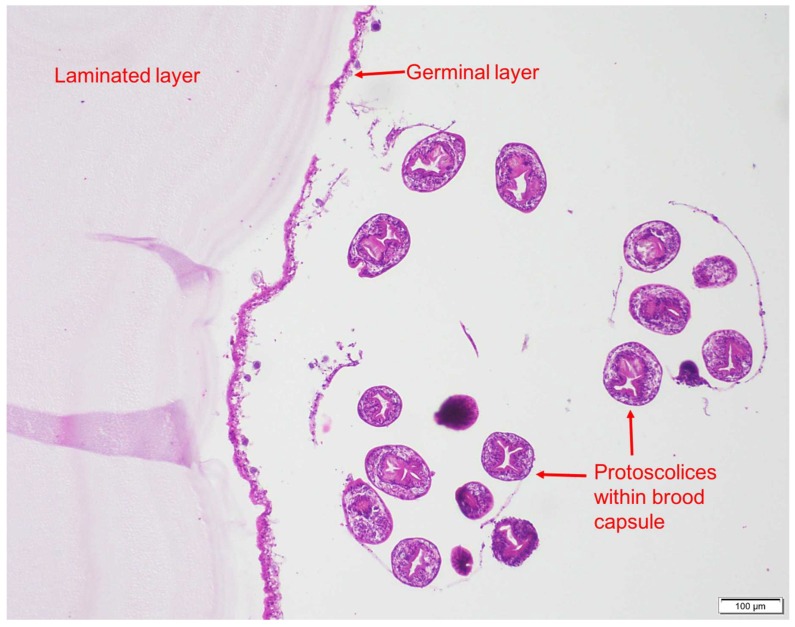
Protoscolices within cyst. Photo: C. Constantinoiu*.*

Multiple active (and fertile) *E. granulosus* hydatid cysts and cysts in various stages of degeneration were found on histopathology. Histopathologic examination of lungs revealed a well-formed, thickly encapsulated granuloma, the center of which contained a thick laminated hyaline membrane, surrounded by a thin rim of neutrophils, macrophages, and lymphocytes, consistent with a hydatid cyst. The liver showed a section of cyst wall containing numerous (up to ten) protoscolices within brood capsules (Figure 3).

### 2.2. Case #2

The second case of *E. granulosus* in a Lumholtz’s tree-kangaroo was an 8.5 kg adult male. Based on weight, pelage coloration and dental wear, the animal was estimated to be greater than six years old. The animal had been found in an emaciated, obtundate state on the side of a road. Clinical assessment showed 3–5% dehydration (based on skin turgor); a phthisical left globe with irregular surface to the cornea and an opaque right eye with what appeared to be a mature cataract. There was an area of moist, erosive dermatitis extending from the right inguinal region along the caudomedial aspect of the right rear limb with a 30 × 60 mm granulomatous lesion at the cranial aspect of the limb. A presumption of vehicular trauma was made, and the animal was euthanized due to age, condition, and poor prognosis.

**Figure 4:**
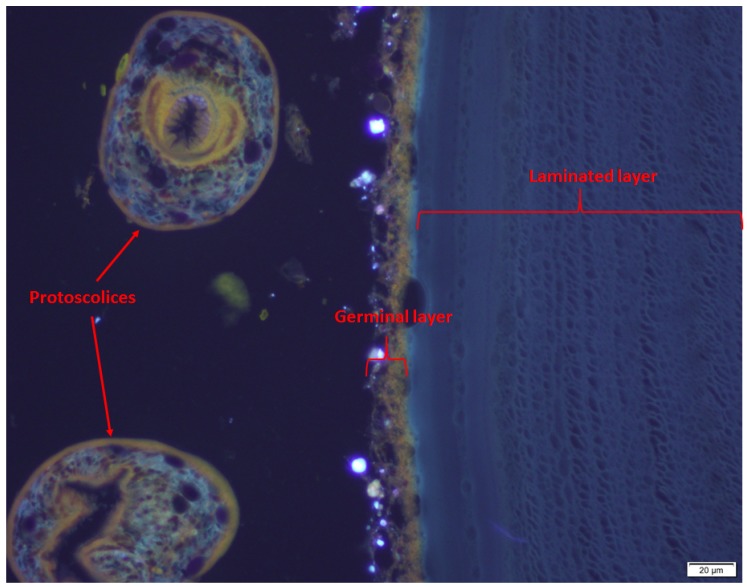
Showing protoscolices, germinal layer and laminated layer’ photo credit: C. Constantinouiu.

**Figure 5:**
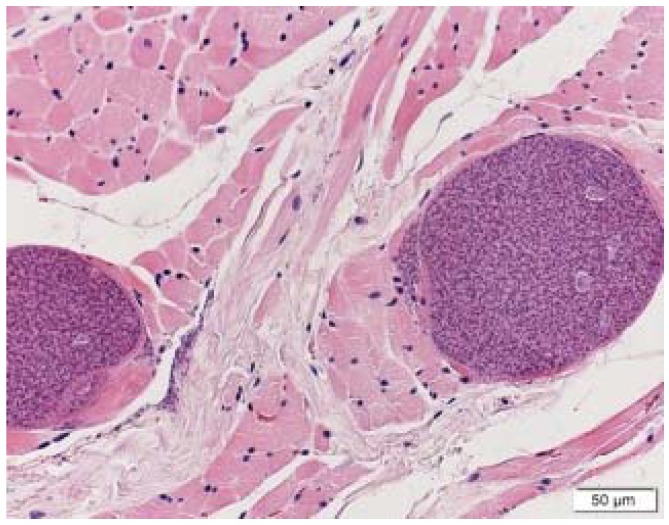
Section of tongue containing presumptive sarcocysts. Photo: L. Johnson.

On gross post-mortem examination, multiple hydatid cysts were found in the liver and lungs. Some had degenerate cysts which lacked a germinal membrane, others had viable protoscolices. The cyst membranes were unusually thick. They were surrounded by a fibroblastic response with giant cells, indicating chronic inflammation. Calcarous bodies were noted in the laminated layers and the germinal layers were thick (Figure 4). The lungs showed evidence of bacterial pneumonia as well as an eosinophilic response to the cysts. Multiple presumptive sarcocysts, previously reported in Lumholtz’s tree-kangaroo by Speare [13], were seen in the tongue (Figure 5).

## 3. Discussion and Results

Historically, *E. granulosus* (s.l.) was considered a serious and potentially fatal health threat in Australia [14]. Significant control measures were instituted, particularly in Tasmania, where the disease was subsequently eradicated. *E. granulosus* (s.l.) G1 is the only strain of echinococcus identified in Australia [15]. PCR on the samples from these two cases would have been useful to determine the strain of *E. granulosus* (s.l.); however, due to resource constraints, this was not done. The inability to eliminate the disease on the mainland has been due to the presence of a wildlife reservoir, lack of political interest and funding for disease eradication, and lack of requirements for reporting the disease in livestock and humans [16]. Hydatid disease has been demonstrated to cause depression in growth rates of some species of livestock [17]. Infection with hydatid cysts in cattle represents a significant economic loss to the beef industry in Queensland north of the Tropic of Capricorn [17]. There is no surveillance program in abattoirs for monitoring hydatidosis in livestock. Since 2008, human echinococcosis has been a non-notifiable disease in Queensland [18], yet hydatidosis is still a potentially-significant human health risk in Australia, with approximately 80–100 human cases diagnosed each year [4,19]. In humans, cystic echinococcosis can have a long period of asymptomatic incubation before the cysts grow large enough to cause one or more of the following: a fracture, abdominal pain, hepatomegaly or, if the cysts burst, anaphylaxis [20,21].

The Atherton Tablelands is an agricultural area of increasing human population, growing from 26,320 in 1976 to 44,350 in 2007, with considerable interface between forest and scrub, pasture for mainly cattle, farmland and residential areas [22,23]. Finding tree-kangaroos, a species that does not spend much of its time on the ground, infected with hydatid cysts may indicate that the abundance of dogs (either dingoes, feral or domestic dogs) infected with *E. granulosus* (s.l.) on the Atherton Tablelands is relatively high and increasing and therefore, an under-estimated zoonotic risk. There is considerable beef and dairy production as well as a growing human population, especially in semi-rural town areas in the region. *E. granulosus* may have relatively high transmission rates on the Atherton Tablelands as eggs would remain viable in the environment for a year under favorable conditions (shady, cool, damp) [4]. *E. granulosus* eggs in canid faeces are immediately infective to humans and coprophagous flies can be an important conduit for spreading infection by feeding on dingo/wild dog faeces and then moving onto human food [11,16]. Encroachment of dingoes into urban areas as well as the increasing use of domestic dogs for pig-hunting may be increasing the risk of transmission of *E. granulosus* to humans in urban and rural residential regions [6,16,24,25].

This study reports detection of *E. granulosus* cysts through post-mortem examination of Lumholtz’s tree-kangaroos as part of a wildlife health surveillance PhD project. Macropods, including tree-kangaroos, are commonly identified by wildlife carers as having ‘pneumonia’; they may actually have compromised lung function due to hydatid cysts or other infectious diseases (e.g., toxoplasmosis) [26,27,28]. Macropods entering the wildlife care network may not be examined by a veterinarian and few receive post-mortem examination, so quantifying the disease in sylvatic hosts is extremely difficult especially as attempts to develop serologic tests to diagnose hydatidosis in live animals have been unsuccessful [5,29]. Nevertheless, a one health surveillance program tailored to the Atherton Tablelands for hydatid disease via post-mortem examinations of wildlife, surveying for *Echinococcus* coproantigens in dog faeces and surveillance of livers and lungs of cattle at abattoirs may be useful in determining the prevalence and risk of transmission of *E. granulosus* (s.l.) in the region. This would enable practical measures to be undertaken to reduce the risk of human infection and the incidence of the disease in wildlife and domestic stock. 

## Supplementary Materials

The following are available online at http://www.mdpi.com/2414-6366/3/2/47/s1, Figure S1: Male Lumholtz’s tree-kangaroo. Photo by J. Hopkinson.

## References

[1] Atkinson J.A.M., Gray D.J., Clements A.C.A., Barnes T.S., McManus D.P., Yang Y.R. (2013). Environmental changes impacting *Echinococcus* transmission: Research to support predictive surveillance and control. Glob. Chang. Biol..

[2] Bristow B.N., Lee S., Shafir S., Sorvillo F. (2012). Human echinococcosis mortality in the United States, 1990-2007. PLoS Negl. Trop. Dis..

[3] Torgerson P.R., Macpherson C.N.L. (2011). The socioeconomic burden of parasitic zoonoses: Global trends. Vet. Parasitol..

[4] Jenkins D.J., Macpherson C.N.L. (2003). Transmission ecology of *Echinococcus* in wildlife in Australia and Africa. Parasitology.

[5] Barnes T.S., Deplazes P., Gottstein B., Jenkins D.J., Mathis A., Siles-Lucas M., Torgerson P.R., Ziadinov I., Heath D.D. (2012). Challenges for diagnosis and control of cystic hydatid disease. Acta Trop..

[6] Banks D.J.D., Copeman D.B., Skerratt L.F. (2006). *Echinococcus granulosus* in northern Queensland: 2. Ecological determinants of infection in beef cattle. Aust. Vet. J..

[7] Jenkins D.J., Morris B. (2003). *Echinococcus granulosus* in wildlife in and around the Kosciuszko National Park, south-eastern Australia. Aust. Vet. J..

[8] Barnes T.S., Hinds L.A., Jenkins D.J., Bielefeldt-Ohmann H., Lightowlers M.W., Coleman G.T. (2011). Comparative pathology of pulmonary hydatid cysts in macropods and sheep. J. Comp. Pathol..

[9] Barnes T.S., Goldizen A.W., Morton J.M., Coleman G.T. (2008). Cystic echinococcosis in a wild population of the brush-tailed rock-wallaby (*Petrogale penicillata*), a threatened macropodid. Parasitology.

[10] Newell G.R. (1999). Australia’s tree-kangaroos: Current issues in their conservation. Biol. Conserv..

[11] Lawson J.R., Gemmell M.A. (1990). Transmission of taeniid tapeworm eggs via blowflies to intermediate hosts. Parasitology.

[12] Vernes K., Dennis A., Winter J. (2001). Mammalian diet and broad hunting strategy of the dingo (*Canis familiaris dingo*) in the wet tropical rain forests of northeastern Australia. Biotropica.

[13] Speare R., Donovan J.A., Thomas A.D., Speare P.J., Grigg G., Jarman P., Hume I. (1989). Diseases of free-ranging Macropodoidea. Kangaroos, Wallabies and Rat-Kangaroos.

[14] Gemmell M.A. (1990). Australasian contributions to an understanding of the epidemiology and control of hydatid disease caused by *Echinococcus granulosus*—Past, present and future. Int. J. Parasitol..

[15] Eckert J., World Health Organization (2001). WHO/OIE Manual on Echinococcosis in Humans and Animals: A Public Health Problem of Global Concern.

[16] Jenkins D.J., Allen L., Goullet M. (2008). Encroachment of *Echinococcus granulosus* into urban areas in eastern Queensland, Australia. Aust. Vet. J..

[17] Banks D.J.D., Copeman D.B., Skerratt L.F., Molina E.C. (2006). *Echinococcus granulosus* in northern Queensland: 1. Prevalence in cattle. Aust. Vet. J..

[18] Queensland Government Health Conditions: Category: Infections and Parasites: Hydatid Disease. http://conditions.health.qld.gov.au/HealthCondition/condition/14/165/81/Hydatid-Disease.

[19] Carmena D., Cardona G.A. (2014). Echinococcosis in wild carnivorous species: Epidemiology, genotypic diversity, and implications for veterinary public health. Vet. Parasitol..

[20] Mandal S., Deb Mandal M. (2012). Human cystic echinococcosis: Epidemiologic, zoonotic, clinical, diagnostic and therapeutic aspects. Asian Pac. J. Trop. Med..

[21] WHO WHO *Echinococcus* Fact Sheet. http://www.who.int/mediacentre/factsheets/fs377/en/.

[22] QGPIFU Far North Queensland Region: A Demographic Profile. http://www.dilgp.qld.gov.au/resources/plan/far-north-queensland/background/demographic-report-final.pdf.

[23] QGSD Far North Queensland Region. http://www.statedevelopment.qld.gov.au/resources/factsheet/regional/far-north-queensland-region-fact-sheet.pdf.

[24] Jenkins D.J., McKinlay A., Duolong H.E., Bradshaw H., Craig P.S. (2006). Detection of *Echinococcus granulosus* coproantigens in faeces from naturally infected rural domestic dogs in south eastern Australia. Aust. Vet. J..

[25] Jenkins D.J., Lievaart J.J., Boufana B., Lett W.S., Bradshaw H., Armua-Fernandez M.T. (2014). *Echinococcus granulosus* and other intestinal helminths: Current status of prevalence and management in rural dogs of eastern Australia. Aust. Vet. J..

[26] Jackson S.M.E.C. (2003). Australian Mammals: Biology and Captive Management.

[27] Staker L. (2006). The Complete Guide to the Care of Macropods.

[28] Vogelnest L., Woods R. (2008). Medicine of Australian Mammals.

[29] Barnes T.S., Li J., Coleman G.T., McManus D.P. (2008). Development and evaluation of immunoblot-based serodiagnostic tests for hydatid infection in macropodids. J. Wildl. Dis..

