# Supplementary material for: Echinococcus Granulosus Infection in Two Free-Ranging Lumholtz’s Tree-Kangaroo (Dendrolagus lumholtzi) from the Atherton Tablelands, Queensland
Source: Trop Med Infect Dis. 2018 May 3;3(2):47. doi: 10.3390/tropicalmed3020047 (PMC6073813; doi:10.3390/tropicalmed3020047)
Supplement: Supplementary file 1 [file tropicalmed-03-00047-s001.pdf]

Tropical Medicine and Infectious Disease

MDPI

Supplementary Materials: Echinococcus Granulosus Infection in Two Free-Ranging Lumholtz'sTree-Kangaroo (*Dendrolagus lumholtzi*) from the Atherton Tablelands, Queensland

Amy L. Shima, Constantin C. Constantinoiu, Linda K. Johnson and Lee F. Skerratt

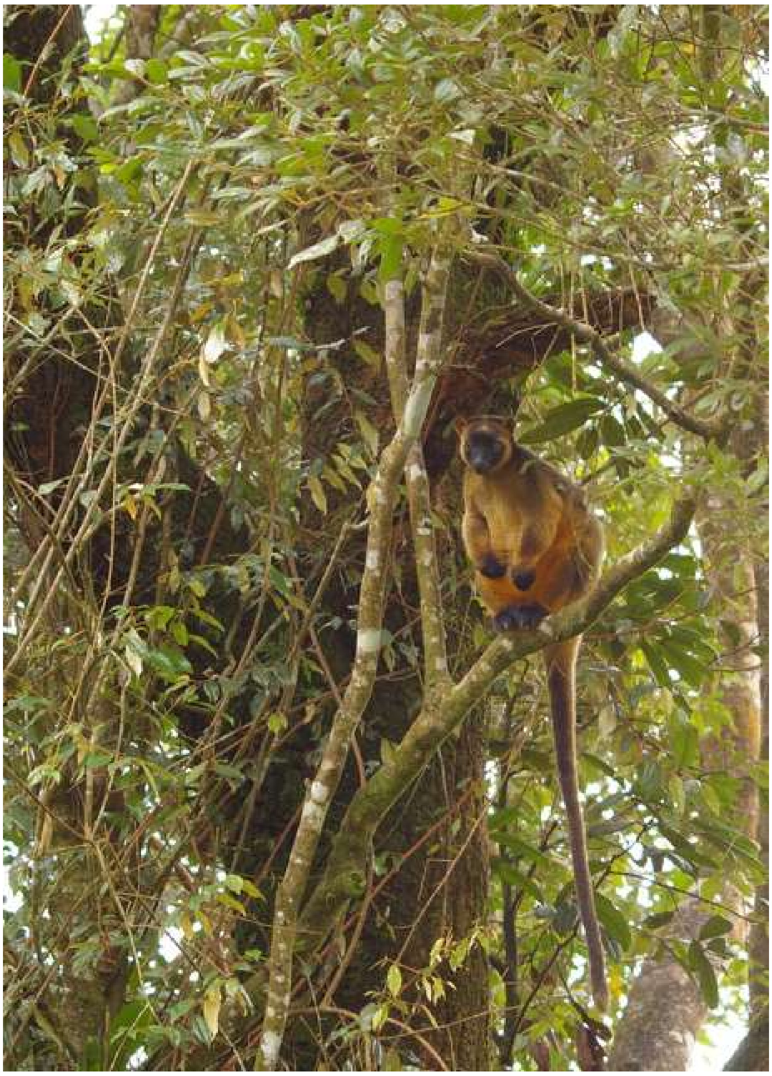

Figure S1: Male Lumholtz's tree-kangaroo. Photo by J. Hopkinson.
